# What is the impact of genetic transformation on wheat allergenicity?

**DOI:** 10.1186/2045-7022-5-S3-P133

**Published:** 2015-03-30

**Authors:** Roberta Lupi, Stefania Masci, Florence Pineau, Gilbert Deshayes, Denise-Anne Moneret-Vautrin, Olivier Tranquet, Sandra Denery-Papini, Colette Larré

**Affiliations:** 1 Institut National de la Recherche Agronomique, UR1268 BIA, Nantes, France; 2 Universita degli Studi della Tuscia, DAFNE, Viterbo, Italy; 3 Faculté de Medecine de Nancy, Service d’ Allergologie, Centre Hospitalier Jean Monnet, Epinal, France

## 

Wheat allergy occurs by inhalation (baker's asthma) and ingestion (food allergy), but may also develop by contact in some cases. The responsible allergens of wheat are proteins accounting for about 10-15% of the grain dry weight. Wheat proteins are divided into two groups: the salt soluble fraction (albumins/globulins) and the gluten proteins (gliadins and glutenins). This latter group is responsible for celiac disease and also for food allergy, whereas the albumins/globulins are involved in baker's asthma and in some food allergy. Genetic modification (GM) technology for crop improvement has recently emerged and its impact on allergenicity must be evaluated, as recommended by the Codex Alimentarius.

Following this recommendation, we first compared two GM lines with their parents using an allergenomic approach then in a second step we aimed at comparing the amount of allergenic polypeptides in these GM wheats, their untransformed genotypes and those measured among twenty commercial cultivars, either durum or bread wheats.

In order to characterize the accumulation of allergenic proteins in wheats, sera from children and adults with clinically documented wheat allergy were used. The investigation is focused mainly on the soluble protein fraction of wheat.

For the comparison of GM lines with their natural counterparts, 2D immunoblot followed by mass spectrometry analysis and protein identification was set up. ELISA tests were performed on the whole set of genotypes.

Few differences at molecular and quantitative levels were revealed between the GM lines and their counterparts. Two new IgE-binding proteins were detected for one GM line.[1] We also observed that the genetic transformation may impact IgE reactivity, either in a positive or in a negative way.[2]

This study leads us to conclude that a wide variation exists in the amount of allergenic polypeptides among durum and bread wheat cultivars, and that the differences observed between GM wheats and their parents are within the range of these 20 cultivated wheats.
